# Racial disparities in ankylosing spondylitis risk following tonsillectomy: a large-scale retrospective analysis

**DOI:** 10.3389/fimmu.2026.1741434

**Published:** 2026-03-06

**Authors:** Chien-Han Tsao, James Cheng-Chung Wei, Yu-Hsun Wang

**Affiliations:** 1 Department of Otolaryngology, Chung Shan Medical University Hospital, Taichung, Taiwan; 2 School of Medicine, Chung Shan Medical University, Taichung, Taiwan; 3 Institute of Medicine, Chung Shan Medical University, Taichung, Taiwan; 4 Division of Allergy, Immunology and Rheumatology, Chung Shan Medical University Hospital, Taichung, Taiwan; 5 Graduate Institute of Integrated Medicine, China Medical University, Taichung, Taiwan; 6 Department of Medical Research, Chung Shan Medical University Hospital, Taichung, Taiwan

**Keywords:** ankylosing spondylitis, propensity score matching, racial disparities, retrospective cohort study, tonsillectomy

## Abstract

**Objective:**

The objective was to investigate the association between tonsillectomy and subsequent ankylosing spondylitis (AS) risk, with particular emphasis on racial disparities in disease susceptibility.

**Methods:**

A retrospective cohort study was conducted using de-identified electronic health records from approximately 120 million patients in the collaboration network in the United States. Patients diagnosed with tonsillar and adenoidal diseases between 2005 and 2023 were selected and divided into surgical and non-surgical cohorts. Propensity score matching (PSM) was employed to balance baseline differences and control for confounding factors, and statistical analyses were performed using Kaplan-Meier survival analysis and Cox proportional hazards models.

**Results:**

After PSM, 173,483 individuals were included in each cohort with well-balanced baseline characteristics. Overall, tonsillectomy did not significantly increase AS risk (HR = 1.26, 95% CI: 0.90-1.75, *p* = 0.210). Age- and sex-stratified analyses yielded consistent results. However, race-stratified analysis revealed that White individuals who underwent tonsillectomy had significantly elevated AS risk (HR = 1.80, 95% CI: 1.19-2.72, *p* = 0.005) and higher cumulative incidence compared to matched controls, a finding not observed in other racial groups.

**Conclusion:**

This large-scale study identifies being of White ancestry as a significant effect modifier in the relationship between tonsillectomy and AS development. These findings warrant closer post-operative surveillance for AS symptoms in White patients undergoing tonsillectomy and further mechanistic research.

## Graphical Abstract

**Figure:**
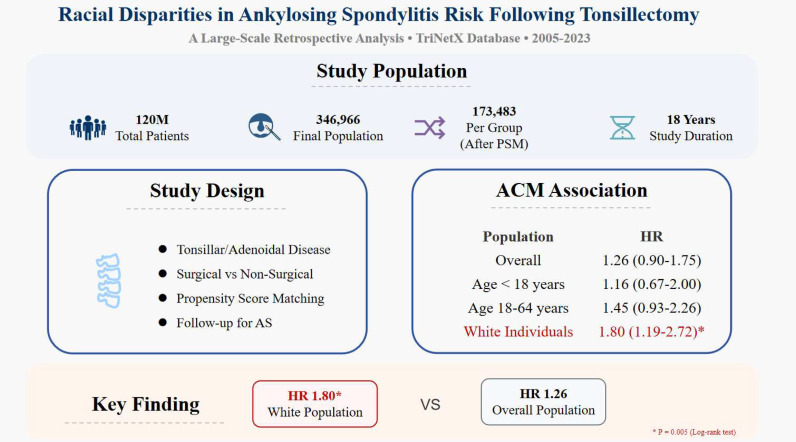

## Introduction

Ankylosing spondylitis (AS) represents a prototypic immune-mediated axial spondyloarthritis characterized by chronic inflammation of spinal entheses, pathological osteoproliferation, and progressive axial ankylosis. With a global prevalence of 0.07-0.32%, AS imposes a substantial socioeconomic burden through chronic disability and reduced quality of life (1, 2). Despite advances in understanding AS pathogenesis, the complex interplay between genetic susceptibility, environmental triggers, and immune dysregulation remains incompletely elucidated (3–5). Genome-wide association studies (GWAS) have identified over 100 risk loci, with HLA-B27 demonstrating the strongest association (present in 85-95% of AS patients) through mechanisms involving aberrant peptide presentation, endoplasmic reticulum stress responses, and IL-23/Th17 axis activation (6). Notably, HLA-B27 prevalence exhibits marked variation based on ancestry, being most common in populations of European descent (8-14%) compared to African (2-4%) and Asian (2-9%) descent. Beyond genetic factors, environmental determinants, including smoking exposure, occupational mechanical stress, vitamin D insufficiency, and obesity-mediated adipokine dysregulation, have been implicated in AS pathogenesis (7–10).

Emerging evidence underscores the gut-joint axis in AS pathogenesis, wherein intestinal dysbiosis may prime pathogenic immune responses (11). Similarly, the oropharyngeal immune system has gained increasing attention, with epidemiological studies suggesting associations between recurrent tonsillitis and subsequent AS development (12). This observation raises critical questions about how surgical manipulation of pharyngeal lymphoid tissue may influence long-term autoimmune risk.

Currently, tonsillectomy is one of the most common surgical procedures performed on children in the United States, primarily to address adenotonsillar hypertrophy (13). While tonsillectomy theoretically modulates regional immunity by removing chronically inflamed lymphoid tissue, its long-term immunological consequences remain paradoxical, potentially eliminating pathogenic antigen exposure while disrupting pharyngeal microbiome homeostasis (14, 15). A national case-control study reported that a history of tonsillectomy in childhood (adjusted OR 1.38; 95% CI 1.06 to 1.77) is associated with an increased risk of AS (16). However, this study did not take racial factors into consideration.

Importantly, the hallmark of AS—progressive spinal ankylosis—reflects more than immune activation: inflammation at entheseal and peri-entheseal sites can initiate a stromal repair program that culminates in pathological osteogenesis. In the conceptual framework of enthesitis, an initial phase of mechanosensation and immune activation triggered by mechanical and/or infectious stress is followed by innate inflammatory responses and IL-17/IL-22–driven mesenchymal proliferation of resident mesenchymal stem/stromal cells from the peri-entheseal periosteum, ultimately promoting entheseal chondrogenesis/osteogenesis and pathological new bone formation (17). Moreover, mechanical strain–induced mechanotransduction, potentially mediated by mechanosensitive ion channels such as Piezo1, may further amplify the coupling between inflammation and new bone formation, for which targeted therapy remains limited once structural damage is established (9, 18). Together, these mechanisms provide a biologic framework linking mucosal immune perturbations to ectopic bone formation. Although big-data studies have limited explanatory power with respect to molecular mechanisms, we hypothesized that excluding potential prodromal (antecedent) factors might yield different findings.

Building upon prior nationwide register-based studies from Taiwan and Sweden (e.g., Chao and Lindström) (12, 16), we conducted a large-scale retrospective cohort study using a large, multi-racial electronic health record database to evaluate the association between tonsillectomy and incident ankylosing spondylitis and to examine potential heterogeneity by race. Race-stratified analyses and effect-modification assessment were prespecified given known differences in genetic predisposition and immune profiles across populations. Accordingly, the novelty of the present work lies primarily in validating and extending prior observations by testing generalizability and quantifying racial and ethnic heterogeneity in a large multi-ethnic database, rather than claiming a first discovery.

## Method

### Data source

This retrospective cohort study analyzed de-identified electronic health records (EHRs) from the U.S.-based federated health research network aggregating data from approximately 120 million patients across 92 healthcare organizations. The database encompasses comprehensive clinical information, including demographics, diagnoses (ICD-10-CM), medications (RxNorm/ATC), procedures (CPT-4/ICD-10-PCS/SNOMED), and laboratory results (LOINC) from diverse healthcare settings, including academic medical centers, community hospitals, and specialty clinics (19).

This study was exempted from informed consent requirements under Section §164.514(a) of the Health Insurance Portability and Accountability Act (HIPAA) Privacy Rule, as it utilized de-identified secondary data without direct human subject interaction. The de-identification process adhered to HIPAA §164.514(b) (1) standards, verified by qualified experts. Ethical approval was granted by the Institutional Review Board of Chung Shan Medical University Hospital (IRB No.: CS2-21176).

### Study design and participants

**Figure 1:**
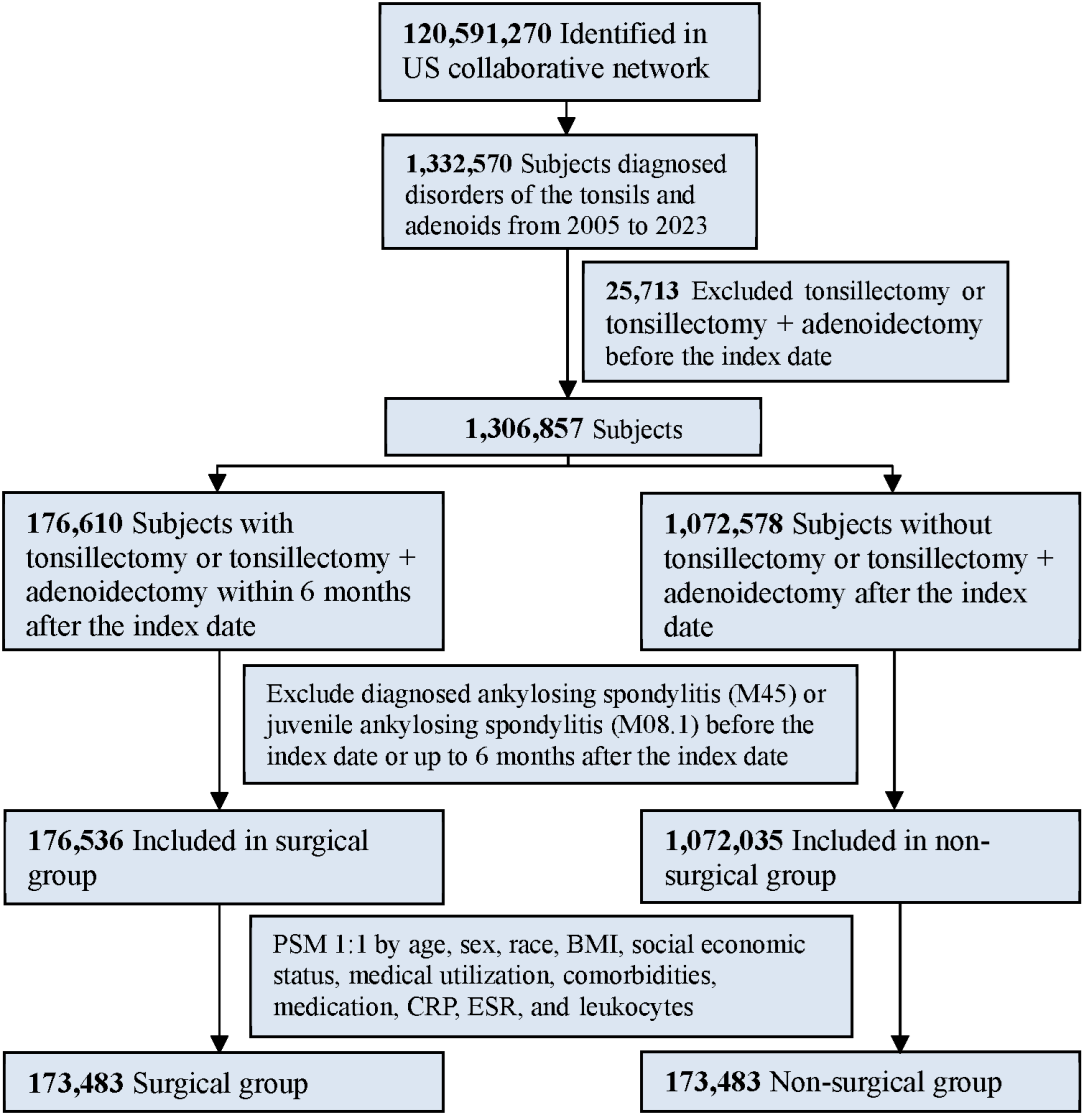
Flow-chart of subject selection.

**Figure 1** illustrates the cohort construction flowchart. The study population comprised individuals diagnosed with tonsillar and adenoidal disorders between 1 January 2005 and 31 December 2023, including streptococcal tonsillitis (ICD-10-CM: J03.0), chronic tonsillitis and adenoiditis (ICD-10-CM: J35.0), tonsillar hypertrophy (ICD-10-CM: J35.1), and peritonsillar abscess (ICD-10-CM: J36). The index date was defined as the first recorded diagnosis of any qualifying condition.

Participants were classified into two cohorts. The surgical cohort included patients who underwent tonsillectomy or tonsillectomy + adenoidectomy within 6 months following the index date (procedure codes detailed in **Supplementary Table 1**). The non-surgical cohort comprised patients who did not receive these procedures after the index date. The following exclusion criteria were applied to both cohorts. First, individuals with a prior tonsillectomy or tonsillectomy + adenoidectomy before the index date were excluded. Second, individuals with a diagnosis of AS (ICD-10-CM: M45) or juvenile AS (ICD-10-CM: M08.1) recorded before or within 6 months after the index date were excluded. This 6-month exclusion window was implemented to minimize reverse causation bias, ensure incident AS cases, and avoid immortal time bias. In the primary analysis, we defined the exposure as tonsillectomy with or without adenoidectomy performed within 6 months after the index date, aiming to capture an “early tonsillectomy” window that provides clearer clinical contrast between groups. Because the timing of surgery may influence both cohort composition and observed effect estimates, we additionally conducted a sensitivity analysis by extending the exposure ascertainment window to 12 months after the index date to evaluate the impact of the time window on the association of tonsillectomy with incident ankylosing spondylitis. The 12-month analysis was performed using the same propensity score matching and outcome modeling strategy as the main analysis, and the results are reported in the **Supplementary Material** (**Supplementary Table 3**). We also note that when treatment timing varies, methodological frameworks using time-dependent propensity scores and risk-set matching based on Cox models have been proposed to address time-varying treatment assignment (20), and we cite this approach as a relevant methodological context for interpreting timing-related sensitivity analyses.

### Baseline variables and covariates

Comprehensive baseline data were extracted from medical records covering the 12-month period preceding the index date, encompassing the following: first, demographics, including age, sex, race and ethnicity, body mass index (BMI), and socioeconomic status indicators; second, healthcare utilization, including ambulatory visits, emergency department encounters, and inpatient admissions; third, comorbidities, including nicotine dependence, alcohol-related disorders, overweight and obesity, hypertension, hyperlipidemia, diabetes mellitus, asthma, allergic rhinitis, atopic dermatitis, chronic/acute sinusitis, obstructive sleep apnea, and Sjögren’s syndrome; fourth, medications, including corticosteroids for systemic use, non-steroidal anti-inflammatory drugs, and antibacterials for systemic use; fifth, laboratory markers, including C-reactive protein (CRP), erythrocyte sedimentation rate (ESR), leukocyte counts. However, within the TriNetX analytic platform, individual-level patterns of missing laboratory data and the exact proportions of missing values for specific laboratory variables cannot be directly assessed. Detailed diagnostic and procedural codes are provided in **Supplementary Table 2**.

### Propensity score matching

To minimize confounding and selection bias, 1:1 propensity score matching (PSM) was performed using a greedy nearest-neighbor algorithm with a caliper width of 0.1 pooled standard deviations. Propensity score matching was performed using complete-case analysis on the predefined baseline covariates that were reliably available within the platform. Individuals with missing values for any of the variables included in the matching algorithm were excluded automatically by the TriNetX matching procedure. The propensity score model incorporated all baseline variables mentioned above to estimate the probability of receiving surgical intervention. Balance between matched cohorts was assessed using standardized mean differences (SMD), with SMD < 0.1 indicating adequate balance. Patients in both cohorts were followed from 6 months post-index date until the first occurrence of AS diagnosis. Patients were censored at the time of their last recorded clinical encounter within the TriNetX network.

### Statistical analysis

All statistical analyses were conducted using the network Analytics platform (integrating R version 4.0.2). The primary outcome was incident AS diagnosis. Kaplan-Meier survival analysis was employed to estimate cumulative AS incidence in surgical versus non-surgical cohorts, with between-group differences assessed via log-rank tests. Cox proportional hazards regression models estimated hazard ratios (HRs) with 95% confidence intervals (CIs) for AS risk, adjusting for residual confounding through the matched design. Pre-specified stratified analyses examined age (<18 years, 18–64 years), sex (male, female), and race (White, Black, Asian, other). In addition, tonsillar and adenoidal disorders were classified by surgical indication into non-inflammatory conditions (tonsillar hypertrophy; ICD-10-CM: J35.1) and inflammatory conditions (streptococcal tonsillitis, chronic tonsillitis and adenoiditis, and peritonsillar abscess; ICD-10-CM: J03.0, J35.0, and J36). The proportional hazards assumption was evaluated using Schoenfeld residuals. Statistical significance was set at two-tailed p < 0.05. Given the exploratory nature of stratified analyses, no adjustment for multiple comparisons was applied, and results should be interpreted accordingly. This study has been reported in line with the STROBE criteria (21).

## Result

### Cohort characteristics and propensity score matching

After applying inclusion and exclusion criteria, 1,248,571 patients were identified (176,536 surgical candidates and 1,072,035 non-surgical patients). Following 1:1 PSM, each cohort comprised 173,483 individuals with excellent baseline balance (all SMDs < 0.1, **Table 1**). Pre-matching disparities were notable for age (SMD > 0.2) and obstructive sleep apnea (SMD > 0.15), which were effectively balanced post-matching.

**Table 1:** Demographic characteristics of the surgical group and the non-surgical group.

Characteristic	Before PSM		SMD	After PSM		SMD
	Surgical group N = 176536	Non-Surgical group N = 1072035		Surgical group N = 173483	Non-Surgical group N = 173483	
Age, Mean ± SD	12.45 ± 12.10	18.38 ± 16.55	**0.409**	12.56 ± 12.17	12.77 ± 12.49	0.017
Sex						
Female	93672 (53.06)	572797 (53.43)	0.007	92283 (53.19)	92294 (53.20)	<0.001
Male	78882 (44.68)	470797 (43.92)	0.015	77229 (44.52)	77142 (44.47)	0.001
Unknown	3982 (2.26)	28441 (2.65)	0.026	3971 (2.29)	4047 (2.33)	0.003
Race						
White	110650 (62.68)	638381 (59.55)	0.064	108783 (62.71)	109239 (62.97)	0.005
Black or African American	22163 (12.55)	160756 (15.00)	0.071	21707 (12.51)	20650 (11.90)	0.019
Asian	3471 (1.97)	28617 (2.67)	0.047	3442 (1.98)	3413 (1.97)	0.001
Native Hawaiian or Other Pacific Islander	399 (0.23)	3615 (0.34)	0.021	393 (0.23)	390 (0.23)	<0.001
American Indian or Alaska Native	579 (0.33)	4953 (0.46)	0.021	577 (0.33)	570 (0.33)	0.001
Other Race	10379 (5.88)	65562 (6.12)	0.010	10177 (5.87)	10289 (5.93)	0.003
Unknown Race	28895 (16.37)	170151 (15.87)	0.013	28404 (16.37)	28932 (16.68)	0.008
BMI, Mean ± SD	23.95 ± 8.45	25.70 ± 8.77	0.203	24.01 ± 8.45	23.97 ± 8.80	0.005
Social economic status						
Persons with potential health hazards related to socioeconomic and psychosocial circumstances	1670 (0.95)	9634 (0.90)	0.005	1585 (0.91)	1492 (0.86)	0.006
Housing/economic circumstances problem	171 (0.10)	1403 (0.13)	0.010	169 (0.10)	171 (0.10)	<0.001
Problems related to education and literacy	176 (0.10)	1045 (0.10)	0.001	167 (0.10)	177 (0.10)	0.002
Employment or unemployment problems	29 (0.02)	376 (0.04)	0.012	29 (0.02)	27 (0.02)	0.001
Occupational exposure to risk factors	24 (0.01)	243 (0.02)	0.007	24 (0.01)	25 (0.01)	<0.001
Medical utilization						
Ambulatory	103301 (58.52)	630043 (58.77)	0.005	100624 (58.00)	99747 (57.50)	0.010
Emergency	25645 (14.53)	184530 (17.21)	0.074	25188 (14.52)	23451 (13.52)	0.029
Inpatient Encounter	8865 (5.02)	46853 (4.37)	0.031	8425 (4.86)	8172 (4.71)	0.007
Comorbidities						
Nicotine dependence	1691 (0.96)	19094 (1.78)	0.071	1689 (0.97)	1630 (0.94)	0.003
Alcohol related disorders	263 (0.15)	3467 (0.32)	0.036	262 (0.15)	239 (0.14)	0.003
Overweight and obesity	7897 (4.47)	47473 (4.43)	0.002	7549 (4.35)	7270 (4.19)	0.008
Hypertension	3481 (1.97)	43074 (4.02)	0.120	3430 (1.98)	3482 (2.01)	0.002
Hyperlipidemia	2899 (1.64)	37563 (3.50)	0.118	2856 (1.65)	2844 (1.64)	0.001
Diabetes mellitus	1468 (0.83)	19358 (1.81)	0.085	1459 (0.84)	1470 (0.85)	0.001
Asthma	12569 (7.12)	66609 (6.21)	0.036	12046 (6.94)	11341 (6.54)	0.016
Allergic rhinitis	9866 (5.59)	50080 (4.67)	0.042	9480 (5.47)	8635 (4.98)	0.022
Atopic dermatitis	2534 (1.44)	12547 (1.17)	0.023	2450 (1.41)	2163 (1.25)	0.014
Chronic sinusitis	5247 (2.97)	22937 (2.14)	0.053	5135 (2.96)	4493 (2.59)	0.023
Acute sinusitis	4918 (2.79)	37770 (3.52)	0.042	4849 (2.80)	4251 (2.45)	0.022
Obstructive sleep apnea	16381 (9.28)	25405 (2.37)	**0.298**	13329 (7.68)	13907 (8.02)	0.012
Sjögren syndrome	51 (0.03)	600 (0.06)	0.013	50 (0.03)	54 (0.03)	0.001
Medications						
Corticosteroids for systemic use	29569 (16.75)	156362 (14.59)	0.060	28767 (16.58)	27500 (15.85)	0.020
Non-steroidal anti-inflammatory drugs	24611 (13.94)	151321 (14.12)	0.005	24094 (13.89)	22737 (13.11)	0.023
Antibacterial for systemic use	51883 (29.39)	307246 (28.66)	0.016	50886 (29.33)	48586 (28.01)	0.029
Laboratory						
C-reactive protein (mg/L)	23.67 ± 40.32	24.47 ± 44.81	0.019	23.66 ± 40.36	25.80 ± 42.98	0.051
Erythrocyte sedimentation rate (mm/h)	17.60 ± 17.85	18.99 ± 20.12	0.073	17.50 ± 17.66	18.36 ± 19.45	0.046
Leukocytes [#/volume] in Blood (10*3/uL)	9.97 ± 74.55	11.61 ± 117.01	0.017	10.00 ± 75.59	11.37 ± 108.73	0.015

SMD, standardized mean difference. Bold values indicate baseline imbalance between groups (absolute SMD ≥ 0.1).

The mean age in the surgical cohort was 12.56 years (SD ± 12.17) compared to 12.77 years (SD ± 12.49) in the non-surgical cohort. Female individuals constituted 53.2% of the total sample. Racial distribution was as follows: White (67.8%), Black (15.3%), Asian (3.2%), and other races (13.7%). The baseline prevalence of inflammatory markers was low and comparable between groups, with CRP elevation (>10 mg/L) in <2% and ESR elevation in <3% of both cohorts.

### Overall ankylosing spondylitis risk

**Figure 2:**
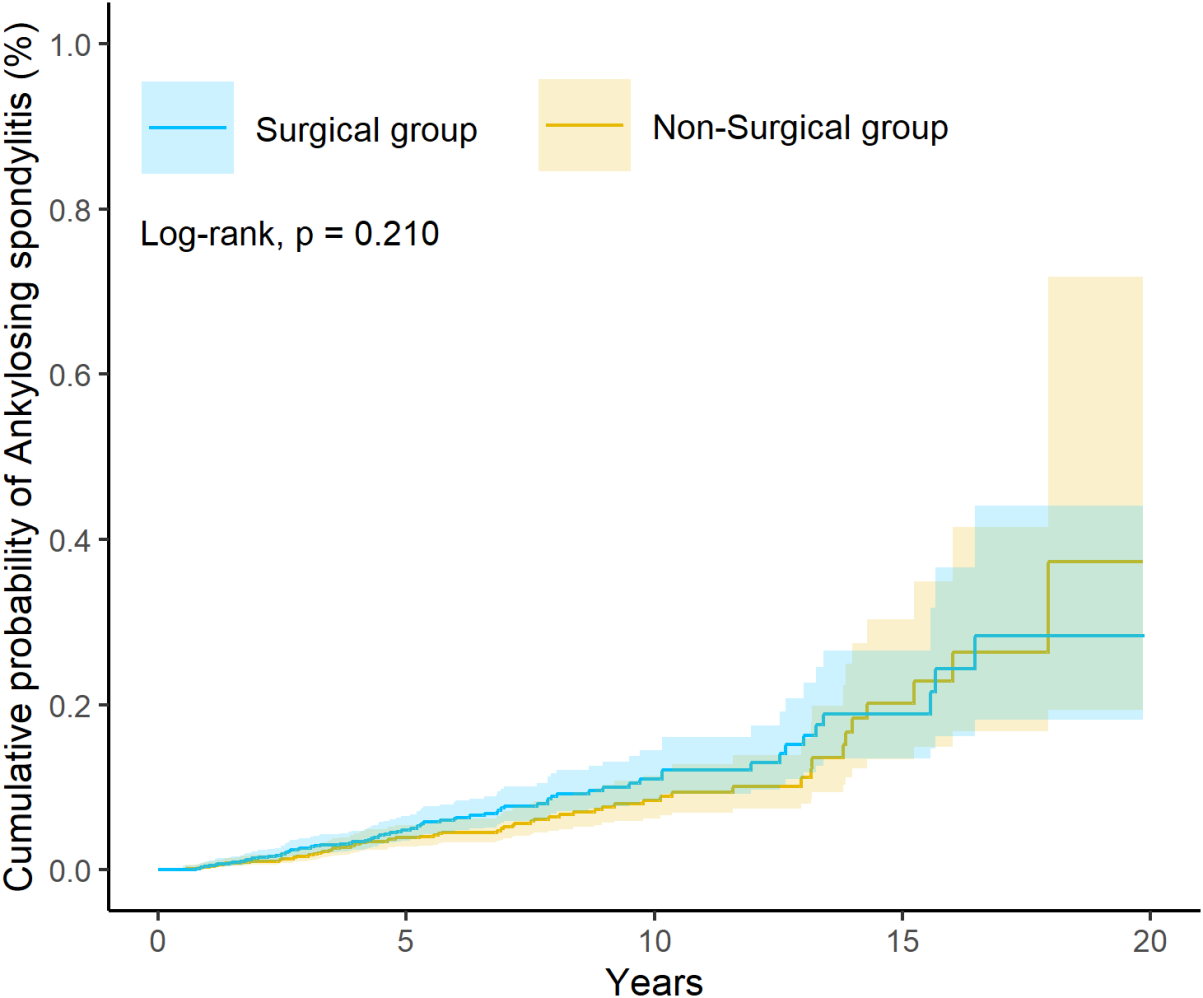
Kaplan-Meier plot for risk of ankylosing spondylitis.

The median follow-up duration was 3.1 years (interquartile range [IQR], 6.2 years) in the surgical group and 3.4 years (IQR, 6.3 years) in the non-surgical group. Incident ankylosing spondylitis occurred in 76 patients (0.044%) in the surgical cohort and 64 patients (0.037%) in the non-surgical cohort. Cox regression analysis revealed no significant overall increase in AS risk following tonsillectomy (HR = 1.26, 95% CI: 0.90-1.75, *p* = 0.210, **Table 2**). Kaplan-Meier survival analysis similarly demonstrated no significant between-group difference in cumulative AS incidence (log-rank test *p* = 0.210, **Figure 2**). In the sensitivity analysis, the surgical group exhibited a similar risk of ankylosing spondylitis compared with the non-surgical group (HR = 1.22, 95% CI: 0.92–1.62; **Supplementary Table 3**).

**Table 2:** Risk of ankylosing spondylitis exposed to the surgical group compared to the non-surgical group.

Outcome	No. of event (Paired N = 173483)		HR (95% C.I.)	P for proportional hazards assumption
	Surgical group	Non-Surgical group		
Ankylosing spondylitis	76	64	1.26 (0.90–1.75)	0.225

### Stratified analyses by age, sex, and race

Age-stratified analyses showed no significant AS risk elevation in pediatric (<18 years: HR = 1.16, 95% CI: 0.67-2.00) or adult (18–64 years: HR = 1.45, 95% CI: 0.93-2.26) surgical cohorts (**Table 3**). Similarly, sex-stratified analyses revealed no significant associations in female (HR = 1.31, 95% CI: 0.80-2.14) or male (HR = 1.22, 95% CI: 0.76-1.95) individuals.

**Table 3:** Subgroup analysis of risk of ankylosing spondylitis exposed to the surgical group compared to the non-surgical group.

Subgroup	Surgical group		Non-Surgical group			P for proportional hazards assumption
	N	No. of event	N	No. of event	HR (95% C.I.)	
Age						
<18	126447	27	126447	25	1.16 (0.67–2.00)	0.886
18-64	41813	47	41813	33	1.45 (0.93–2.26)	0.496
≥65	593	10	593	0	N/A	N/A
Sex						
Female	92363	50	92363	48	1.10 (0.74–1.63)	0.868
Male	76926	26	76926	23	1.21 (0.69–2.12)	0.883
Race						
White	108105	61	108105	35	1.80 (1.19–2.72)	0.939
Black or African American	21419	10	21419	10	0.86 (0.26–2.81)	0.272
Asian	3433	10	3433	10	2.52 (0.23–27.88)	0.100
Tonsillar and adenoidal disorders						
Non-inflammatory	89252	30	89252	32	1.02 (0.62–1.68)	0.483
Inflammatory	85084	45	85084	44	1.11 (0.73–1.69)	0.494

If the patient’s count is 1-10, the results indicate a count of 10.

N/A, Not applicable.

Notably, race-stratified analysis revealed significant heterogeneity in treatment effects. White individuals who underwent tonsillectomy demonstrated substantially elevated AS risk (HR = 1.80, 95% CI: 1.19-2.72, *p* = 0.005) compared to matched non-surgical White controls. This association was specific to White patients and not observed in Black (HR = 0.85, 95% CI: 0.32-2.26), Asian (HR = 1.02, 95% CI: 0.14-7.38), or other racial groups (HR = 1.15, 95% CI: 0.45-2.91). In the sensitivity analysis, a significantly higher risk of ankylosing spondylitis was observed among White individuals in the surgical group compared with the non-surgical group (HR = 1.68, 95% CI: 1.18–2.39; **Supplementary Table 3**). Furthermore, when tonsillar and adenoidal disorders were further classified into non-inflammatory and inflammatory indications, no significantly increased risk of ankylosing spondylitis was observed among patients who underwent tonsillectomy compared with the non-surgical group in either stratum.

**Figure 3:**
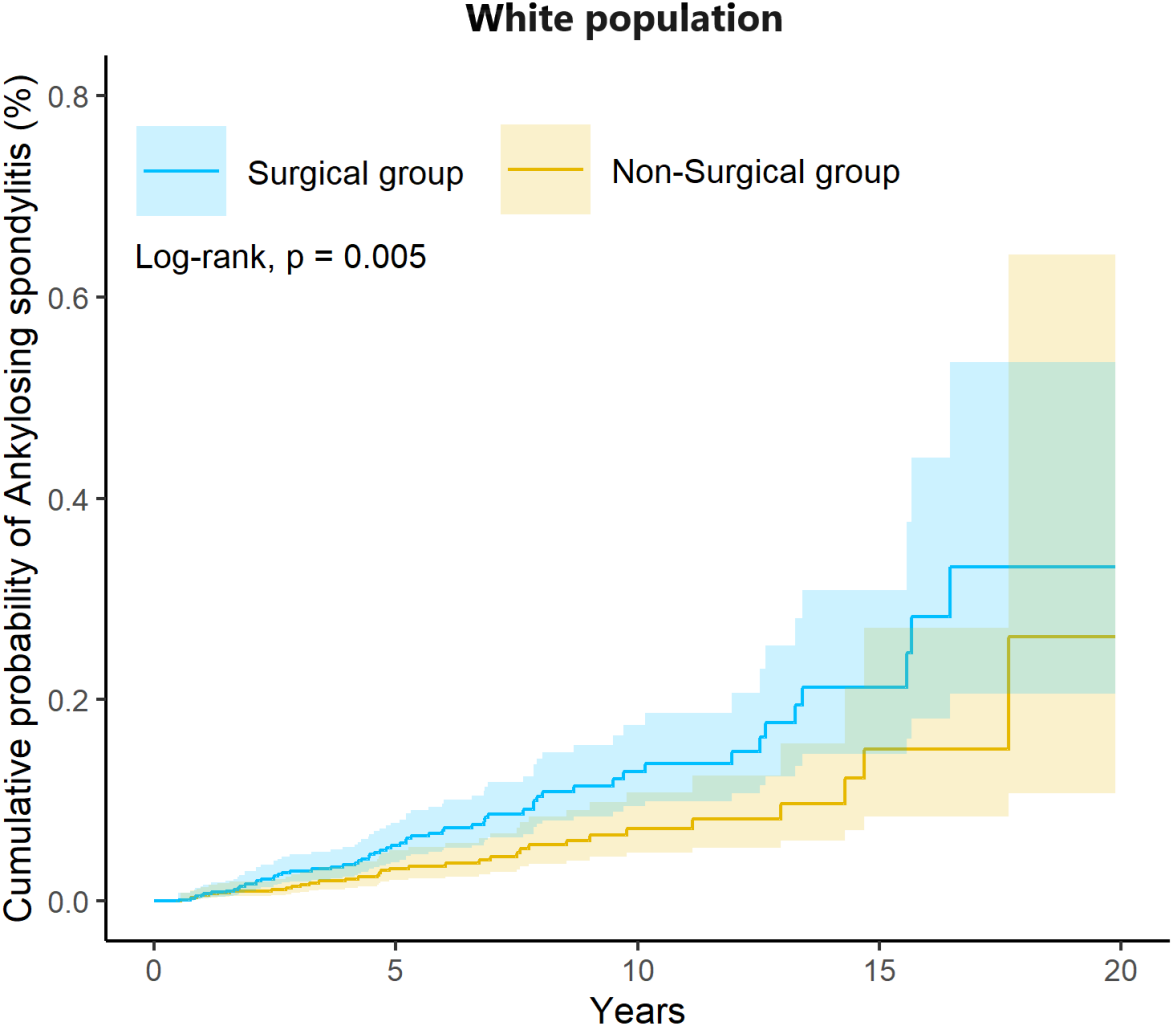
Kaplan-Meier plot illustrating the risk of Ankylosing Spondylitis in the White population.

Kaplan-Meier curves for White participants confirmed significantly higher cumulative AS incidence in the surgical versus non-surgical cohort (5-year incidence: 0.089% vs. 0.048%, log-rank test p = 0.005, **Figure 3**).

## Discussion

This large retrospective matched cohort study, including 346,966 matched individuals, examined the association between tonsillectomy and the risk of ankylosing spondylitis (AS) and identified race as an important effect modifier. Although no overall association was detected in the full cohort, White patients who underwent tonsillectomy had an 80% higher risk of subsequent AS (HR = 1.80). This observed racial heterogeneity may have clinically meaningful implications.

In interpreting our findings, it is important to distinguish between tonsillitis (infectious/inflammatory exposure) and tonsillectomy (surgical removal), as these are related but biologically distinct exposures and may not exert the same effects on immune-mediated outcomes. Accordingly, we revised our literature comparison to align prior evidence with the specific exposure definition used in the present study.

With respect to tonsillectomy, Swedish register-based evidence reported an increased risk of AS following childhood tonsillectomy. Lindström et al. observed an adjusted odds ratio (OR) of 1.38 (95% CI, 1.06–1.77) in a national study, and Morin et al. similarly reported an OR of 1.30 (95% CI, 1.13–1.49) in a nationwide case–control analysis, with the association remaining evident in a sibling-comparison design (16, 22). Both studies suggested that the association was strongest when tonsillectomy occurred between ages 5 and 9 years, although neither performed race-stratified analyses. Given that Swedish and broader Scandinavian populations are predominantly White and have a relatively high prevalence of HLA-B27, our work extends this literature by leveraging a multi-ethnic database to directly evaluate racial and ethnic heterogeneity. Notably, the magnitude of association observed among White patients in our cohort was directionally consistent with these Scandinavian findings, and differences in effect size should be interpreted cautiously given differences in study design, outcome definitions, and effect measures (HR vs OR), as well as potential differences in surgical indications and healthcare systems. Overall, our results should be interpreted as a validation and extension of existing evidence rather than a first discovery and warrant replication in independent multi-ethnic cohorts.

In contrast, prior studies, such as that by Chao et al., primarily evaluated tonsillitis rather than tonsillectomy, suggesting that recurrent tonsillar inflammation may itself be associated with later AS risk (12). This distinction is clinically relevant because the underlying indication leading to surgery (e.g., recurrent infection vs hypertrophy/OSA) may confound observed associations and could plausibly modify the direction or magnitude of risk. Evidence from other immune-mediated diseases (e.g., IgA nephropathy) further illustrates that infection-related tonsillar inflammation and tonsillectomy can play contrasting roles; therefore, we avoid treating tonsillitis and tonsillectomy as interchangeable exposures and interpret our findings in the context of the surgical exposure definition and indication-related confounding.

Although HLA-B27 prevalence is higher among the White population, prior studies have suggested that the comorbidity burden of AS may be disproportionately high among African American individuals in the United States (23), and other work has likewise documented racial differences in AS epidemiology and/or clinical features (24). Given the substantial racial/ethnic heterogeneity of the U.S. population, rigorously characterizing racial differences in AS is therefore of critical importance. On this basis, we incorporated race-stratified analyses into our study design and prespecified the hypothesis that the association of interest may differ across racial groups.

Building upon prior work (12, 16), we conducted a large-scale retrospective cohort study using a collaborative network database to: first, investigate the association between tonsillectomy and AS risk in a diverse U.S. population; second, perform comprehensive stratified analyses by age, sex, and race to identify potentially high-risk subgroups; and third, explore potential mechanisms underlying any observed racial disparities. Given well-described racial differences in HLA-B27 prevalence and AS phenotype/severity (25, 26), we pre-specified race-stratified analyses to evaluate potential effect modification and to minimize the risk that population-level averages obscure subgroup associations. We hypothesized that tonsillectomy-associated AS risk would vary by race, potentially reflecting differences in genetic predisposition and immune system reactivity.

Our null findings in the overall cohort, despite adequate statistical power (173,483 per arm), may reflect several factors: first, racial diversity in our U.S.-based cohort (32.2% were not White) diluting race-specific associations; second, differences in surgical indications and techniques between U.S. and European healthcare systems; and third, more rigorous confounder adjustment through comprehensive PSM including 30+ baseline variables. Importantly, when we restricted analysis to White participants—demographically comparable to the Scandinavian studies—we observed a directionally consistent association among White participants compared with Scandinavian studies, although direct comparison of effect size should be made cautiously given different effect measures (HR vs OR) and study designs.

The observed racial specificity of tonsillectomy-associated AS risk likely reflects complex gene-environment interactions. First, genetic predisposition plays a central role: HLA-B27 prevalence in White populations substantially exceeds that in Black and Asian populations (27). Given that 85-95% of AS patients are HLA-B27 positive, the larger pool of genetically susceptible individuals in White populations may amplify tonsillectomy-triggered autoimmune responses. The HLA-B27 molecule’s tendency toward misfolding and endoplasmic reticulum stress could be exacerbated by post-surgical immune dysregulation. Second, tonsillectomy may differentially impact immune regulation across racial groups. Tonsils shape T-cell repertoires and maintain oral-pharyngeal immune homeostasis (28, 29). Surgical removal disrupts this immunological “education center,” potentially unleashing autoreactive T-cell clones in genetically predisposed individuals. Racial differences in tonsillar immune architecture, microbial colonization, and cytokine profiles may render White individuals more susceptible to post-surgical IL-23/Th17 axis activation—the canonical pathway in AS pathogenesis (30, 31). Third, environmental and epigenetic factors may contribute. White populations demonstrate distinct DNA methylation patterns in immune-related genes, potentially modifying responses to surgical immune perturbation (3, 32). Childhood infection patterns, antibiotic exposure, and microbiome composition—which vary by race —may interact with HLA-B27 status to influence AS susceptibility (3). Finally, healthcare disparities warrant consideration. Racial differences in tonsillectomy indications, surgical timing, and perioperative care could contribute to observed associations. White children may undergo tonsillectomy at younger ages or for different indications, potentially coinciding with critical windows of immune system development (33). In addition to the role of tonsillectomy, the underlying tonsillar pathology itself may influence the baseline risk of developing ankylosing spondylitis. Different tonsillar conditions, particularly inflammatory versus non-inflammatory disorders, may reflect distinct immune environments and systemic inflammatory burdens that could independently affect autoimmune disease susceptibility. The present study was not designed to evaluate the association between tonsillar pathology and AS risk independent of tonsillectomy, such as through analyses censoring individuals at the time of surgery. Consequently, potential differences in baseline AS risk across tonsillar pathologies could not be directly assessed.

Our findings have several clinical implications. First, they suggest potential value in closer post-operative surveillance for AS symptoms (inflammatory back pain, morning stiffness, enthesitis) in White patients undergoing childhood tonsillectomy, particularly those with a family history of spondyloarthropathy or known HLA-B27 positivity. Second, these results highlight the importance of considering genetic background in surgical decision-making for borderline indications, potentially favoring watchful waiting or medical management in high-risk individuals. Third, our study underscores the critical need for race-stratified analyses in immunological research. Population-level null findings may mask clinically important subgroup effects, potentially leading to missed opportunities for targeted prevention or surveillance strategies. Future research should first validate our findings in independent cohorts with HLA-B27 genotyping; second, investigate whether AS risk varies by tonsillectomy indication; and third, explore whether prophylactic interventions (e.g., probiotics, microbiome modulation) could mitigate AS risk in high-risk individuals.

Several limitations of this study should be acknowledged. First, the use of the network database introduces inherent drawbacks associated with electronic health record research. The diagnosis of ankylosing spondylitis was based on diagnostic codes, which may lack the accuracy of clinical diagnoses, particularly for early or atypical cases. Second, despite using propensity score matching to control for measured confounding factors, some unidentified or unmeasured confounders, such as environmental exposures and lifestyle factors not recorded in the database, may still affect the accuracy of the study results. Importantly, genetic susceptibility represents a major source of potential confounding. The database does not contain genetic information, including HLA-B27 genotype, which is a well-established and strong risk factor for ankylosing spondylitis and whose prevalence differs substantially across racial groups. Variations in the distribution of HLA-B27 and other genetic risk factors may partially account for the observed race-specific associations and should be considered when interpreting the findings. In addition, body mass index (BMI), C-reactive protein (CRP), erythrocyte sedimentation rate (ESR), and socioeconomic indicators are incompletely captured in the TriNetX network. Consequently, residual confounding due to unavailable or unmeasured variables cannot be excluded. In addition, because the timing of tonsillectomy may influence cohort composition and effect estimates, we conducted a sensitivity analysis extending the exposure ascertainment window from 6 to 12 months after the index date, and the findings were generally consistent (**Supplementary Table 3**). Nevertheless, alternative approaches such as risk-set matching using a time-dependent propensity score (20) could further address time-varying treatment assignment in future work. Third, although tonsillar and adenoidal disorders were stratified by inflammatory versus non-inflammatory indications, formal statistical testing of interaction terms between underlying tonsillar pathology and tonsillectomy was considered but could not be performed. Such modeling options, including interaction term testing, are not readily supported within the TriNetX analytic framework used in this study; therefore, we were unable to formally test interaction effects or report corresponding p-values. Fourth, the TriNetX database does not provide sufficient procedural detail to distinguish between partial (intracapsular) and total (extracapsular) tonsillectomy, nor does it capture information on specific surgical techniques. As a result, we were unable to evaluate whether the extent of tonsillar removal or the surgical approach differentially influences the risk of ankylosing spondylitis. Fifth, an additional limitation relates to the age distribution of the study population. The mean age at cohort entry was approximately 12 years, which is substantially younger than the typical age of ankylosing spondylitis onset, generally occurring between 20 and 40 years. This age structure reflects the real-world clinical context in which tonsillectomy is predominantly performed during childhood or adolescence. Consequently, the relatively young cohort and limited follow-up into adulthood may have resulted in a low observed incidence of ankylosing spondylitis. **Sixth**, the database restricts the source population to patients with health insurance who sought medical care during the study period, limiting the generalizability of our findings (34). PSM may help address these limitations. Lastly, data missingness is often unavoidable in electronic health records, potentially leading to bias in the study results. Excluding individuals or indicators with excessive missing values is a viable approach to mitigate this bias.

## Conclusion

This study identified a significantly higher risk of AS in White individuals after tonsillectomy. However, no overall increase in AS risk was observed in the surgical cohort. These findings suggest that the relationship between tonsillectomy and AS may be modified by racial or genetic factors, underscoring the heterogeneity of immune-mediated disease risk across populations. Future studies integrating genetic susceptibility markers, such as HLA-B27 status, and detailed clinical indications for tonsillectomy are warranted to clarify the underlying immunological mechanisms. Additionally, validation in diverse populations and prospective study designs may help determine whether tonsillectomy serves as a context-dependent risk factor for AS rather than a universal trigger.

## Data Availability

The raw data supporting the conclusions of this article will be made available by the authors, without undue reservation.

## Supplementary material

Supplementary Table 1 Code for tonsillectomy or tonsillectomy + adenoidectomy. CPT, Current Procedural Terminology; SNOMED, Systematic Nomenclature of Medicine.

Supplementary Table 2 Code for study variables. ICD-10-CM, International Classification of Diseases, Tenth Revision, Clinical Modification; ATC, Anatomical Therapeutic Chemical; TNX, TrinetX curated.

Supplementary Table 3 Sensitivity analysis for risk of ankylosing spondylitis. The surgical cohort included patients who underwent tonsillectomy or tonsillectomy + adenoidectomy within one year following the index date.

## Glossary

AS: Ankylosing Spondylitis
ATC: Anatomical Therapeutic Chemical Classification System
BMI: Body Mass Index
CI: Confidence Interval
CIs: Confidence Intervals
CPT-4: Current Procedural Terminology 4^th^ edition
CRP: C-Reactive Protein
EHRs: Electronic Health Records
ESR: Erythrocyte Sedimentation Rate
GWAS: Genome-Wide Association Studies
HIPAA: Health Insurance Portability and Accountability Act
HLA: Human Leukocyte Antigen
HR: Hazard Ratio
HRs: Hazard Ratios
ICD-10-CM: International Classification of Diseases 10th Revision Clinical Modification
ICD-10-PCS: International Classification of Diseases 10th Revision Procedure Coding System
IL: Interleukin
IQR: Interquartile Range
IRB: Institutional Review Board
LOINC: Logical Observation Identifiers Names and Codes
OR: Odds Ratio
PSM: Propensity Score Matching
RxNorm: Standardized Naming System for Medications
SD: Standard Deviation
SMD: Standardized Mean Difference
SNOMED: Systematized Nomenclature of Medicine
STROBE: Strengthening the Reporting of Observational Studies in Epidemiology
Th17: T Helper 17 cells.
