# Supplementary material for: Racial disparities in ankylosing spondylitis risk following tonsillectomy: a large-scale retrospective analysis
Source: Front Immunol. 2026 Mar 6;17:1741434. doi: 10.3389/fimmu.2026.1741434 (PMC13002431; doi:10.3389/fimmu.2026.1741434)
Supplement: Supplementary Table 1 — Code for tonsillectomy or tonsillectomy + adenoidectomy. CPT, Current Procedural Terminology; SNOMED, Systematic Nomenclature of Medicine. [file Table1.docx]

Supplementary Table 1 Code for tonsillectomy or tonsillectomy + adenoidectomy

| Name | Code |
| --- | --- |
| Tonsillectomy and adenoidectomy | CPT:1007178 |
| Tonsillectomy | SNOMED:173422009 |
| Tonsillectomy and adenoidectomy | CPT:1007178 |
| Tonsillectomy and adenoidectomy; younger than age 12 | CPT:42820 |
| Tonsillectomy, primary or secondary | CPT:1007181 |
| Tonsillectomy, primary or secondary; age 12 or over | CPT:42826 |
| Tonsillectomy and adenoidectomy; age 12 or over | CPT:42821 |
| Tonsillectomy, primary or secondary; younger than age 12 | CPT:42825 |
| Tonsillectomy and adenoidectomy | SNOMED:28913000 |
| Adenoidectomy, primary | CPT:1007184 |
| Adenoidectomy, primary; younger than age 12 | CPT:42830 |
| Adenoidectomy, primary; age 12 or over | CPT:42831 |
| Adenoidectomy, secondary | CPT:1007187 |
| Adenoidectomy, secondary; younger than age 12 | CPT:42835 |
| Adenoidectomy, secondary; age 12 or over | CPT:42836 |
| Tonsillectomy and adenoidectomy | CPT:1007178 |
| Tonsillectomy and adenoidectomy; younger than age 12 | CPT:42820 |
| Tonsillectomy and adenoidectomy; age 12 or over | CPT:42821 |
| Tonsillectomy and adenoidectomy | SNOMED:28913000 |

CPT: Current Procedural Terminology.

SNOMED: Systematic Nomenclature of Medicine.

Supplementary Table 2 Code for study variables

|  | ICD-10-CM |
| --- | --- |
| Social economic status |  |
| Persons with potential health hazards related to socioeconomic and psychosocial  circumstances | Z55-Z65 |
| Housing/economic circumstances problem | Z59 |
| Problems related to education and literacy | Z55 |
| Employment or unemployment problems | Z56 |
| Occupational exposure to risk factors | Z57 |
| Comorbidities |  |
| Nicotine dependence | F17 |
| Alcohol related disorders | F10 |
| Overweight and obesity | E66 |
| Hypertension | I10 |
| Hyperlipidemia | E78 |
| Diabetes mellitus | E08-E13 |
| Asthma | J45 |
| Allergic rhinitis | J30.9 |
| Atopic dermatitis | L20 |
| Chronic sinusitis | J32 |
| Acute sinusitis | J01 |
| Obstructive sleep apnea | G47.33 |
| Sjögren syndrome | M35.0 |
| Medications |  |
| Corticosteroids for systemic use | ATC: H02 |
| Non-Steroidal Anti-Inflammatory Drugs | ATC: M01A |
| Antibacterial for systemic use | ATC: J01 |
| Laboratory |  |
| C reactive protein (mg/L) | TNX: 9063 |
| Erythrocyte sedimentation rate (mm/h) | TNX:9066 |
| Leukocytes [#/volume] in Blood (10*3/uL) | TNX:9015 |

ICD-10-CM: International Classification of Diseases, Tenth Revision, Clinical Modification.

ATC: Anatomical Therapeutic Chemical.

TNX: TrinetX curated.

**Supplementary Table 3 Sensitivity analysis for risk of ankylosing spondylitis**

|  | Surgical group | | Non-Surgical group | |  | P for proportional hazards assumption |
| --- | --- | --- | --- | --- | --- | --- |
|  | N | No. of event | N | No. of event | HR (95% C.I.) |  |
| All | 194284 | 100 | 194284 | 89 | 1.22 (0.92–1.62) | 0.874 |
| Race: White | 125695 | 80 | 125695 | 51 | 1.68 (1.18–2.39) | 0.392 |

Note: The surgical cohort included patients who underwent tonsillectomy or tonsillectomy + adenoidectomy within one year following the index date
